# The Adult Changes in Thought (ACT) Medical Records Abstraction Project: A Resource for Research on Biological, Psychosocial and Behavioral Factors on the Aging Brain and Alzheimer’s Disease and Related Dementias

**DOI:** 10.3390/brainsci14111075

**Published:** 2024-10-28

**Authors:** Nicole M. Gatto, Anne Renz, Sarah E. Tom, Mary Lyons, Jennifer A. Macuiba, Tammy S. Dodd, Bonnie K. Lind, Shelly L. Gray, Kelly Meyers, Eric B. Larson, Jennifer C. Nelson, Linda K. McEvoy, Sundary Sankaran, Dustin Key, Jeremiah A. Litondo, Paul K. Crane

**Affiliations:** 1 Kaiser Permanente Washington Health Research Institute, Seattle, WA 98101, USA; anne.d.renz@kp.org (A.R.); mary.k.lyons@kp.org (M.L.); jennifer.a.macuiba@kp.org (J.A.M.); tammydodd78@hotmail.com (T.S.D.); bonnie.k.lind@kp.org (B.K.L.); kelly.p.meyers@kp.org (K.M.); ebl@uw.edu (E.B.L.); jen.nelson@kp.org (J.C.N.); linda.k.mcevoy@kp.org (L.K.M.); sundary.sankaran@kp.org (S.S.); dustin.x.key@kp.org (D.K.); jeremiah.a.litondo@kp.org (J.A.L.); 2 Columbia University Irving Medical Center, New York, NY 10032, USA; st3144@cumc.columbia.edu; 3 School of Pharmacy, University of Washington, Seattle, WA 98102, USA; slgray@uw.edu; 4 School of Medicine, University of Washington, Seattle, WA 98195, USA; pcrane@uw.edu

**Keywords:** chart abstraction, historical data, comorbidities, dementia, Alzheimer’s disease, electronic health record data, chart data

## Abstract

Background: Adult Changes in Thought (ACT), a prospective cohort study, enrolls older adult members of Kaiser Permanente Washington. We describe an ambitious project to abstract medical records facilitating epidemiological investigation. Methods: Abstracted data include medications; laboratory results; women’s health; blood pressure; physical injuries; cardiovascular, neurological, psychiatric and other medical conditions. Results: Of 1419 of 5763 participants with completed abstractions, 1387 (97.7%) were deceased; 602 (42.4%) were diagnosed with Alzheimer’s Disease and Related Dementias; 985 (69.4%) had a brain autopsy. Each participant had an average of 34.3 (SD = 13.4) years of data abstracted. Over 64% had pharmacy data preceding 1977; 87.5% had laboratory data preceding 1988. Stroke, anxiety, depression and confusion during hospitalization were common among participants diagnosed with dementia. Conclusions: Medical records are transformed into data for analyses with outcomes derived from other ACT data. We provide detailed, unparalleled longitudinal clinical data to support a variety of epidemiological research on clinical-pathological correlations.

## 1. Introduction

Prospective cohort studies are the gold standard among observational epidemiology study designs but can be cost prohibitive owing to their large study population sizes and lengthy follow-up time [1,2]. Retrospective studies are thus essential for health conditions such as Alzheimer’s disease and related dementias, in which progression from subjective cognitive decline to a clinical dementia diagnosis occurs over many years. Existing historical records can be an asset for retrospective exposure assessment in cohort studies and provide cost savings [1,3]. Patient medical records are a data source for health information [4]. Prior to 2001 in the United States (US), records predominately resided in paper charts, with just 18% of office-based physicians then having an electronic health records (EHR) system [5], and abstracting from handwritten notes in many cases was required. The era of electronic medical records (EMR)/EHR and growth of natural language processing (NLP) tools [6] have increasingly popularized their use in epidemiologic research.

**Figure 1:**
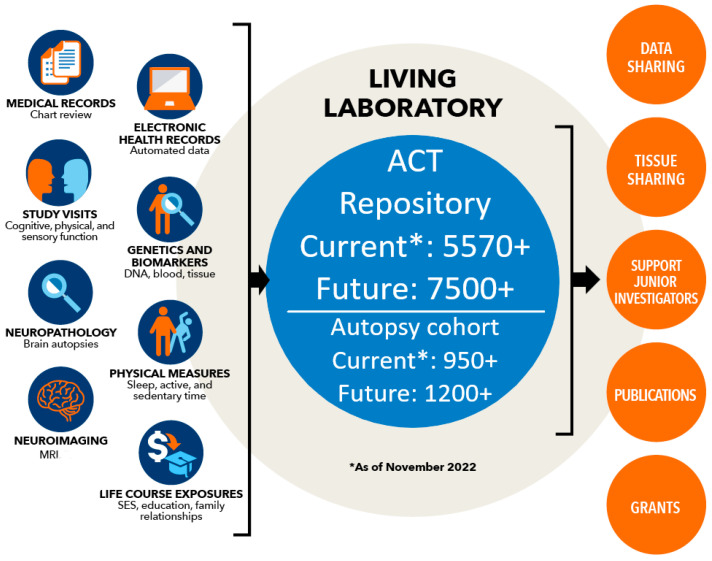
Data sources available for the Adult Changes in Thought (ACT) Study.

When a study cohort is embedded within a healthcare delivery system that provides essentially all care received by its members, unique opportunities may exist to enrich research data with information accessible in the patient’s medical record. Participants in the Adult Changes in Thought (ACT) Study, an ongoing population-based prospective cohort study of dementia among adults 65 years of age and older, receive healthcare services from an integrated healthcare delivery system, some as far back as the 1940s when an innovative Health Care Maintenance organization—Group Health—was formed. In ACT, medical records are available for mid-life exposures prior to cohort enrollment that may be important predictors of brain and general health in later life. Medical chart data have served as complementary sources to other electronic pharmacy, laboratory, enrollment and study visit data in ACT Study research [7,8,9,10,11,12,13,14,15,16,17,18,19]. A schematic representation of data sources available for the ACT cohort, including data abstracted from medical records, is shown in Figure 1.

Here, we describe the development and implementation of a standardized medical record abstraction project to transform extensive paper and electronic medical records from the ACT cohort into high-quality structured data to facilitate analyses. We describe the data collected from chart abstraction and provide demographic characteristics of cohort members for whom abstracted data are currently available. We summarize select medical and health history information for the chart abstraction cohort overall and among those with a dementia diagnosis and with a completed brain autopsy. These data, in conjunction with other ACT Study data, are a valuable resource available to investigators with relevant research questions.

## 2. Materials and Methods

### 2.1. Participants

The ACT study is an ongoing population-based prospective cohort study that began in 1994 [20,21,22] and aims to identify risk and preventive factors for dementia. The study enrolls dementia-free persons aged 65 and older from the membership of Kaiser Permanente Washington [KPWA, previously Group Health Cooperative (GHC)] and follows them longitudinally. KPWA and GHC are pioneers in integrated healthcare delivery systems, providing both medical insurance and healthcare services to members. Medical services are comprehensive and include primary, urgent and specialty care visits, hospitalizations, surgery, and other procedures along with telephone calls and virtual care video visits with healthcare providers.

Between 1994 and 1996, ACT enrolled 2581 GHC members living in the greater Seattle area who were 65 years of age or older, community dwelling, and did not have a diagnosis of dementia at baseline. A second wave of recruitment occurred in 2002–2003 when an expansion cohort of 811 members were enrolled. Beginning in 2004, the study began a continuous enrollment wave to replace participants who died, developed dementia, or withdrew from the study, adding participants annually to maintain an active cohort of approximately 2000 members. This initial continuous enrollment wave was interrupted by the COVID-19 pandemic beginning in March 2020. When recruitment activities were re-started in 2022, ACT began a new initiative emphasizing enrollment of more racially, ethnically and socioeconomically diverse participants. These efforts expanded the geographical recruitment area to include KPWA clinics that serve higher proportions of minoritized groups and involved targeted oversampling to reach greater numbers of diverse older adults. As of the most recent data freeze (25 November 2022), total cumulative enrollment in the ACT study was 5763 people with 49,422 person-years of follow-up (mean 8.6, SD = 6.1 years per person). The active cohort of participants at risk for dementia outcomes was 1664 people, and a total of 985 brain autopsies had been completed. Follow up rates have been consistently high within the ACT cohort. Overall, 12% of participants enrolled ultimately withdrew. The goal of the new diversity-focused recruitment efforts is to expand the active cohort to 3000 people at risk for dementia outcomes with at least 20% of newly enrolled members who identify as American Indian or Alaska Native; Asian American; Black, African American, or African; Hispanic/Latinx; Native Hawaiian or Other Pacific Islander; or another group than non-Hispanic White by April 2026.

### 2.2. ACT Data Collection

At ACT study enrollment, all participants complete an in-person baseline evaluation, which includes the collection of demographic, social, medical history, health behavior, cognitive function, physical function and health status data. Subsequent study visits, either in the study clinic or at the participant’s home, occur about every 2 years until the earliest of either a dementia diagnosis, death, or withdrawal from the study. The Cognitive Abilities Screening Instrument (CASI), a 100-point global cognition assessment, is used to evaluate cognitive function [23]. CASI scores <86 at baseline or biennial visits prompt a standardized diagnostic evaluation, including physical and neurological examinations and administration of a neuropsychological battery. Consensus conferences of study physicians and a clinical psychologist review all these data to ascertain dementia diagnoses [21] using Diagnostic and Statistical Manual of Mental Disorders, fourth edition (DSM-IV) criteria. A diagnosis of probable or possible Alzheimer’s disease dementia is determined based on National Institute of Neurological and Communicative Disorders and Stroke and the Alzheimer’s Disease and Related Disorders Association criteria [24]. Brain autopsies, which assess neuropathologic outcomes, are completed by a board-certified neuropathologist according to published guidelines, who is blinded to the participants’ clinical dementia status [25].

The Kaiser Permanente Interregional Institutional Review Board approved the study (Protocol #RNG211396). Written informed consent is obtained from all ACT study participants, including permission to examine their medical records and, for those who agree, a brain autopsy upon their death.

### 2.3. Overview of Chart Abstraction

ACT chart review and abstraction systematically compiles historical information on study participants to transform medical record data into formats useful for research. Participants are prioritized for chart abstraction in order of (1) deceased with a completed brain autopsy, (2) deceased and previously took insulin for diabetes (as reported in pharmacy records), and (3) in birth order from earliest birth date to most recent. The rationale for this follows. First, neuropathology outcomes are limited to the autopsied cohort, and this prioritization ensures that as many people with autopsy data also have their medical records abstracted. Second, a longstanding interest of ACT investigators is diabetes and metabolism, and electronic pharmacy records do not include information on insulin doses. Finally, prioritizing the earliest birth cohorts facilitates research on the oldest-old while addressing birth cohort effects.

Paper charts for study participants comprised a main source of medical records for chart abstraction prior to the early 2000s. Beginning in 2003, GHC began using EpicCare, an electronic medical record system, which was fully adopted in 2005 when all clinics were documenting medical information entirely online. In addition, different types of medical information (X-rays, lab results, medications) have been scanned into EpicCare and made available for use in ACT.

### 2.4. Chart Abstractors

Research specialists who are rigorously trained and certified follow a defined protocol for abstraction. During an initial training period, 100% of charts abstracted by a new abstractor are re-abstracted by the lead chart abstractor or alternate protocol-certified abstractor. The new abstractor is considered certified in the study protocol when 3 of their abstracted charts are consecutively re-abstracted with ≥93% agreement with the certified abstractor. The new abstractor must achieve 93% agreement with the certified abstractor on the presence/absence of medical conditions/procedures, demographics, social history, and dates and numeric values for labs, weights, and blood pressures. After certification, each protocol certified abstractor participates in an inter-rater reliability (IRR) protocol, which includes full or partial re-abstraction of 5% of completed abstractions by a second abstractor. The goal of the IRR protocol is to identify individual and team training needs, areas where the codebook requires clarification, and any other obstacles in maintaining a high level of abstractor agreement.

### 2.5. Manual of Operations

Chart abstractors utilize a regularly updated 200+ page manual of operations, which is available from the ACT website at https://actagingresearch.org/resources/act-data-repository/medical-record-abstraction-data. The manual is organized in a template format, which includes a short description of the information needed, fields for data entry, a definition of the condition or process to record, any other words or abbreviations used to describe the condition or process, and sources in the medical record where information about the condition or process is commonly found. A discussion section accompanies individual variables and includes additional information needed for decision-making such as how to handle conflicting, missing or unknown elements; prioritization of source documents; reliance on self-reports; establishing chronology; use of specialist records; and variable reports for an item of interest.

### 2.6. Abstracted Data Elements

Decisions about what information to include in the chart review and abstraction protocol were guided by specific aims of the ACT study, investigator research interests, and judged scientific value of the data. Elements to abstract were chosen to maximize utility to the above three aims while maintaining an efficient abstraction process based on elements available in medical records. Decisions were additionally impacted by the ability of chart data to complement other ACT data sources, such as study visit data and non-abstracted EHR claims or diagnosis data.

The over 200 abstracted data elements for each participant include those related to medical and social information on study participants over their entire enrollment in GHC/KPWA. This includes health history; medical conditions and medical procedures (Table 1); female reproductive history and hormone use; laboratory test results preceding 1988 (the start of automated laboratory data); medications preceding 1977 (the start of automated pharmacy data) from categories of: antidepressants, antihypertensives, antipsychotics, diabetes medications including insulin, hormones, sedatives, thyroid medications; surgical history requiring general anesthesia; biometrics and lifestyle factors (including smoking and alcohol intake); as well as demographic characteristics.

### 2.7. Abstraction Process

Abstractors systematically review paper and electronic medical records and abstract specific information with minimal interpretation so that abstracted data reflect exactly what is in the chart. Depending on the type of information, data are abstracted for one time point only, e.g., first adult height and initial diagnoses of chronic conditions such as congestive heart failure (CHF), chronic obstructive pulmonary disease (COPD), diabetes or atrial fibrillation (AFib); yearly, e.g., weight, occurrence of angina, edema, hearing difficulties, hypertension, memory complaints, stroke; or three times per year, e.g., blood pressure (BP), insulin usage, laboratory results including cholesterol and glucose. For paper or electronic charts containing a face page with summary medical information, abstractors verify that these pieces of data are supported elsewhere in the medical record; otherwise, this information is abstracted as notes. Abstractors enter all data directly in an electronic database and complete a check of all required variables to finalize data collection.

### 2.8. Analysis

Descriptive statistics (means ± standard deviations, or medians with interquartile ranges; numbers and percentages) were calculated for selected structured variables collected from the medical record abstraction process. We summarize chart abstracted data according to whether a brain autopsy was performed and by dementia diagnosis status.

## 3. Results

**Figure 2:**
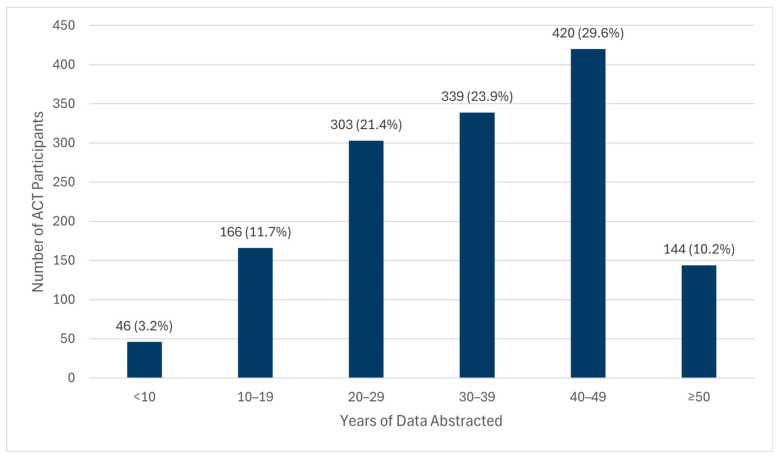
Years of abstracted data for Adult Changes in Thought participants with completed chart review.

Chart abstractions have been completed for 1419 (24.6%) of 5763 ACT participants as of 30 November 2023. Data were abstracted from both paper and electronic sources for 1259 (88.7%) of participants; 146 (10.2%) had exclusively paper and 13 (0.9%) exclusively electronic charts. On average, abstractors spent 13.8 (SD = 12.0) hours to abstract data from charts for each participant. The mean (SD) number of years of abstracted data for a participant was 34.3 (SD = 13.4) (Figure 2). The oldest record reviewed included patient-reported information that dates from 1902.

Of the ACT participants with completed chart abstraction, 1387 (97.7%) were deceased, 985 (69.4%) had a completed brain autopsy, and 602 (42.4%) had received a consensus dementia diagnosis. The average age at ACT study entry of those in the chart abstraction cohort was 77.7 ± 7.1 years; 59.5% were female. Participants had been enrolled in the ACT Study for an average of 11.4 ± 6.1 years. Of those who were deceased, the mean age at death was 89.3 ± 7.0 years. Participants with a dementia diagnosis were slightly older at study entry and at death, had been enrolled in ACT for 1.7 years longer, and a slightly higher proportion were female, compared with the chart abstraction cohort overall (Table 2).

Select clinical characteristics of the chart abstraction cohort are summarized in Table 2. Medical records indicated that several diagnosed conditions were highly prevalent among participants, such as arthritis (89.7%), vision problems (94.8%), hypertension (66.4%) and depression (61.4%). Other conditions were substantially less prevalent in the abstracted record, including Parkinson’s disease (3.7%), diabetes (7.6%) and migraine (8.5%). Most medical conditions were identified at similar rates among those with a brain autopsy or with a dementia diagnosis as the cohort overall (Table 2). Exceptions included stroke, anxiety, depression and confusion during hospitalization, which were more common among participants who developed dementia compared to the overall sample, while cancer and migraine were less common among participants who developed dementia.

Annual records of participant weight and measurements of blood pressure were available for a median of 29 [IQR: 21–36] and 32 [IQR: 24–39] years, respectively. At least one neuroimaging scan was performed on 1004 (70.8%) participants for whom a report was available in the chart. An ejection fraction measurement was available in charts for 976 (68.8%) participants.

Before 1977 and the start of automated pharmacy data, chart abstraction provided records of prescription medications for 64% of participants. A total of 608 different medications were captured by abstracted data, and individual participants had documented use of between one and as many as 49 medications for this period. Abstracted chart data indicated some medications were commonly used by participants, including analgesics such as acetaminophen with codeine (n = 274, 19%), and propoxyphene (n = 300, 21%), the antispasmodic belladonna phenobarbital (n = 249, 18%) and the antihistamine, tripelennamine citrate (n = 93, 7%).

A total of 1242 (87.5%) participants had at least one record from abstracted laboratory data for years preceding 1988, when automated laboratory data became available. These participants had an average number (±SD) of records with lab test values for hemoglobin of 4.7 ± 4.6, creatinine 3.5 ± 3.4, blood calcium 3.1 ± 3.2, glucose 4.8 ± 4.9, and cholesterol 2.0 ± 2.4.

## 4. Discussion

Data abstracted from the medical chart drawn from a healthcare system providing comprehensive, longitudinal care, comprise a robust source of information for observational research studies. KPWA, formerly GHC, has a rich tradition of epidemiologic research based on medical records [26,27]. We showed that it is possible to systematically extract decades’ worth of medical record information into standardized variables for aging research. ACT Study chart abstraction transforms medical records into data ready for analyses with careful attention to quality. Medical records abstraction is designed to complement other sources of high-quality data on this well-characterized cohort, including computerized pharmacy and laboratory data, and research data collected at in-person ACT study visits. Having participants embedded within an integrated health system that retains older paper records in addition to more recent electronic health records facilitates a deeper knowledge of the life course prior to later life brain health outcomes inclusive of dementia. These data can facilitate various investigations where more reliable exposure characterizations can include those deduced from high-quality, longitudinal medical records data rather than a reliance on self-report. Furthermore, and importantly, chart abstracted data extend ACT Study data collection to include information on exposures in midlife that would not otherwise be possible.

Medical chart abstracted data complement the variety of other data that ACT contributes to research on Alzheimer’s disease and related dementias (Figure 1). Compared with studies based on resources such as the Alzheimer’s Disease Neuroimaging Initiative (ADNI), which may be limited in their generalizability owing to ADNI inclusion criteria, ACT contributes population-based data which can overcome such limitations. To this point, we have shown that ACT participants are similar to older adults in the Seattle area enrolled in the Behavioral Risk Factor Surveillance System (BRFSS) [28]. Furthermore, compared with other observational studies of Alzheimer’s disease and related dementias that rely on primary data collection [29,30,31], we are able to supplement our research data collection with information accessible to us from the patient medical record. This strength stems from ACT being embedded within the KPWA healthcare delivery system and helps to reduce the burden of data collection from research study participants. 

Our chart abstracted data demonstrate that many medical conditions were prevalent among ACT participants. Strokes and a history of confusion during hospitalization or of depression were prevalent among participants who died with dementia. It is important to note that these data pertain to a subset of the autopsy cohort, who are a non-random sample of the entire cohort, and analyses should thus consider accounting for possible selection bias [32]. 

ACT’s chart abstraction provides detailed longitudinal clinical data to support a range of topics in epidemiologic studies of the ACT cohort. In addition to epidemiologic research of neurological, neuropathological and neurodegenerative disease outcomes, previous studies have employed ACT chart abstraction data in research on the cohort in the areas of pharmacoepidemiology [11,33], ophthalmology [16] and genetics [14]. Investigations into associations between traumatic brain injury (TBI) and depression [15] and activities of daily living [19], as well as a characterization of neuropathological findings [12] among persons with TBI, utilized chart abstraction data for year and duration of TBI with loss of consciousness for ACT participants across their entire duration of membership in KPWA. Abstracted data on diagnoses were the basis of efforts to validate a Parkinson’s disease case-finding approach in one study of ACT participants [18]. Other studies used chart abstracted data on medications preceding 1977 to complement automated pharmacy data, prescription opioids and non-aspirin nonsteroidal anti-inflammatory drugs (NSAIDs) [10,11] and antihypertensives [33]. Abstracted electrocardiogram (EKG) data were utilized to identify atrial fibrillation and flutter (AF) and to observe persons with permanent AF, using data from at least two EKGs 6 to 36 months apart [34]. Blood pressure measurements abstracted triannually made it possible to ascertain year-to-year variation in systolic BP from age 50 to 90 years by age decade [35]. Among ACT participants with brain autopsy, the combination of chart review and automated laboratory data for fasting glucose and glycated hemoglobin (HbA1c or total glycated hemoglobin) was used to compute the estimated average glucose level for 5-year periods across participants’ lifetimes, covering as recently as the 5 years before death to age periods from 55 to 59 through to 80–84 years [8]. Ancillary studies of subsets of ACT participants focused on vision and hearing rely on chart abstracted data from ophthalmic care [16] and audiological evaluations [17]. 

The goal of the ACT Study chart abstraction project was not to generate a comprehensive dataset derived from the medical record. Rather, the approach has been to anticipate targeted data elements that would be of interest to researchers alongside the prospective data collected from participants. Decisions about what elements to abstract are based on our team’s informed judgment. Consequently, this approach might not provide data to meet every need. Researchers can follow our methods and expand the current chart abstraction to address additional research questions among the ACT cohort with ancillary grant funding. Additionally, promising emerging technologies such as NLP can make it more feasible to extract information from records while reducing the time and labor demands on research staff.

## 5. Conclusions

Data sharing is an aim of the ACT Study, making this rich data resource available to researchers. Data abstracted from medical charts are included in that offering. A particular strength of ACT is our contribution of data to investigations of the life course. Interested investigators can learn more about utilizing ACT data by visiting the ACT Study website: https://actagingresearch.org/.

## Figures and Tables

**Table 1:** Abstracted medical conditions and medical procedures: Adult Changes in Thought (ACT) Study Chart Review and Abstraction.

Automatic Implantable Cardioverter Defibrillator (AICD) Placement ^1^	Epilepsy
Angina	Hearing difficulties
Anxiety	Hypertension
Arthritis	Liver Disease, Chronic or Alcoholic cirrhosis ^1^
Aspirin (ASA) use, chronic	Memory complaints, documentation of
Asthma	Migraine headache
Bipolar illness ^1^	Mini Mental State Examination (MMSE) or Montreal Cognitive Assessment (MOCA), documentation of
Blood transfusion	Myocardial infarction
Coronary Artery Bypass Graft (CABG) procedure	Nephrotic syndrome ^1^
Cancer	Neurological imaging scan of the brain, documentation of
Cardiac arrest	Pacemaker placement and/or removal
Carotid endarterectomy procedure	Parkinson’s disease
Congestive heart failure (CHF) or heart failure (HF)	Physical injuries, fractures and falls
Cholecystectomy procedure	Pneumonia
Confusion during an inpatient hospitalization	Peripheral Vascular Disease (PVD), procedure performed for
Chronic obstructive pulmonary disease (COPD)	Subarachnoid hemorrhage (non-traumatic)
Coronary angioplasty	Subdural hematoma (non-traumatic)
Depression	Valvular heart disease, prosthetic (artificial) valve placement
Diabetes mellitus	Vision problems
Insulin usage	Venous thromboembolism (VTE), deep vein thrombosis (DVT) or pulmonary embolism (PE)
Dialysis, chronic ^1^	Adverse Reaction to Warfarin (Coumadin)
Drug abuse ^1^	Stroke
Edema	Old infarct, date unknown
Ejection Fraction test, documentation of	Transient ischemic attack (TIA)

^1^ no longer abstracting as of 2024.

**Table 2:** Demographic characteristics and select clinical findings of ACT participants with abstracted chart data overall with among those with completed autopsy or dementia diagnosis.

Characteristic	Overall n = 1419 (100%)	With Completed Autopsy n = 985 (69.4%)	With Dementia Diagnosis n = 602 (42.4%)
Age at study entry, years	77.7 ± 7.1	76.7 ± 6.6	78.3 ± 6.8
Age at death, years ^1^	89.3 ± 7.0	89.4 ± 6.6	91.4 ± 5.8
Years enrolled in ACT	11.4 ± 6.1	12.7 ± 6.1	13.1 ± 5.8
Female sex	842 (59.5)	574 (58.3)	382 (63.5)
Medical record data available			
Annual records per participant with			
Weight	29 [21–36]	30 [22–37]	29 [22–37]
Blood pressure	32 [24–39]	33 [25–40]	33 [25–40]
Brain neuroimaging (at least one record)	1004 (70.8)	722 (73.3)	491 (81.6)
Ejection fraction (any record)	976 (68.8); 2 [1–3]	705 (70.0); 2 [1–3]	389 (64.6); 2 [1–3]
Clinical findings (ever diagnosis of)			
Myocardial infarction	556 (39.2)	386 (39.2)	234 (38.9)
Angina	491 (34.6)	328 (33.3)	207 (34.4)
VTE/DVT/PE	178 (12.5)	139 (14.1)	72 (12)
CABG	115 (8.1)	74 (7.5)	48 (8)
Hypertension	942 (66.4)	650 (66)	403 (66.9)
Stroke	693 (48.8)	471 (47.8)	331 (55)
Anxiety	688 (48.5)	464 (47.1)	317 (52.7)
Arthritis	1273 (89.7)	884 (89.7)	548 (91)
Cancer	557 (39.3)	398 (40.4)	187 (31.1)
Cholecystectomy	255 (18)	169 (17.2)	118 (19.6)
Confusion in hospital	490 (34.5)	338 (34.3)	246 (40.9)
COPD	135 (9.5)	101 (10.3)	45 (7.5)
Depression	871 (61.4)	621 (63)	405 (67.3)
Diabetes	108 (7.6)	88 (8.9)	50 (8.3)
Migraine	120 (8.5)	96 (9.7)	40 (6.6)
Parkinson’s disease	53 (3.7)	44 (4.5)	32 (5.3)
Pneumonia	842 (59.3)	592 (60.1)	367 (61)
Vision problems	1345 (94.8)	935 (94.9)	577 (95.8)
Cataracts	1266 (89.2)	884 (89.7)	548 (91)
Cataract surgery	890 (62.7)	626 (63.6)	367 (61)
Glaucoma	289 (20.4)	197 (20)	130 (21.6)
Macular degeneration	491 (34.6)	339 (34.4)	221 (36.7)

^1^ 1387 (97.7%) participants were deceased at time of chart review.

## Data Availability

Data from this analysis cannot be made publicly available for ethical and legal reasons. In order to replicate our findings, a researcher may need access to personal health identifiers (PHI) including dates of birth and death, dates of diagnoses, and ages over 89. These are required variables for the analysis, and we cannot publicly release this information without IRB approval and a Data Use Agreement with interested researchers. However, the datasets used and/or analyzed in the current study are available upon reasonable request and execution of appropriate human subjects review and data sharing agreements by following the process described on the Adult Changes in Thought (ACT) website: actagingresearch.org.

## Abbreviations

Adult Changes in Thought (ACT); Alzheimer’s Disease (AD); Alzheimer’s Disease Neuroimaging Initiative (ADNI); atrial fibrillation (AFib); Automatic implantable cardioverter defibrillator (AICD); Behavioral Risk Factor Surveillance System (BRFSS); blood pressure (BP); Cognitive Abilities Screening Instrument (CASI); Congestive heart failure (CHF); chronic obstructive pulmonary disease (COPD); congestive heart failure (CHF); Coronary Artery Bypass Graft (CABG); deep vein thrombosis (DVT); Diagnostic and Statistical Manual of Mental Disorders, fourth edition (DSM-IV); electronic health records (EHR); electronic medical records (EMR); electrocardiogram (EKG); Group Health Cooperative (GHC); heart failure (HF); inter-rater reliability (IRR); Kaiser Permanente Washington (KPWA); Mini Mental State Examination (MMSE); Montreal Cognitive Assessment (MOCA); natural language processing (NLP); nonsteroidal anti-inflammatory drugs (NSAIDs); Peripheral Vascular Disease (PVD); pulmonary embolism (PE); Transient ischemic attack (TIA); traumatic brain injury (TBI); United States (US); Venous thromboembolism (VTE).
